# Text Topics and Treatment Response in Internet-Delivered Cognitive Behavioral Therapy for Generalized Anxiety Disorder: Text Mining Study

**DOI:** 10.2196/38911

**Published:** 2022-11-09

**Authors:** Sanna Mylläri, Suoma Eeva Saarni, Ville Ritola, Grigori Joffe, Jan-Henry Stenberg, Ole André Solbakken, Nikolai Olavi Czajkowski, Tom Rosenström

**Affiliations:** 1 Department of Psychology and Logopedics Faculty of Medicine University of Helsinki Helsinki Finland; 2 Department of Psychiatry Brain Center Helsinki University Hospital and University of Helsinki Helsinki Finland; 3 Department of Psychology University of Oslo Oslo Norway; 4 Department of Mental Disorders Norwegian Institute of Public Health Oslo Norway

**Keywords:** iCBT, CBT, psychotherapy, internet therapy, anxiety, topic modeling, natural language processing

## Abstract

**Background:**

Text mining methods such as topic modeling can offer valuable information on how and to whom internet-delivered cognitive behavioral therapies (iCBT) work. Although iCBT treatments provide convenient data for topic modeling, it has rarely been used in this context.

**Objective:**

Our aims were to apply topic modeling to written assignment texts from iCBT for generalized anxiety disorder and explore the resulting topics’ associations with treatment response. As predetermining the number of topics presents a considerable challenge in topic modeling, we also aimed to explore a novel method for topic number selection.

**Methods:**

We defined 2 latent Dirichlet allocation (LDA) topic models using a novel data-driven and a more commonly used interpretability-based topic number selection approaches. We used multilevel models to associate the topics with continuous-valued treatment response, defined as the rate of per-session change in GAD-7 sum scores throughout the treatment.

**Results:**

Our analyses included 1686 patients. We observed 2 topics that were associated with better than average treatment response: “well-being of family, pets, and loved ones” from the data-driven LDA model (B=–0.10 SD/session/∆topic; 95% CI –016 to –0.03) and “children, family issues” from the interpretability-based model (B=–0.18 SD/session/∆topic; 95% CI –0.31 to –0.05). Two topics were associated with worse treatment response: “monitoring of thoughts and worries” from the data-driven model (B=0.06 SD/session/∆topic; 95% CI 0.01 to 0.11) and “internet therapy” from the interpretability-based model (B=0.27 SD/session/∆topic; 95% CI 0.07 to 0.46).

**Conclusions:**

The 2 LDA models were different in terms of their interpretability and broadness of topics but both contained topics that were associated with treatment response in an interpretable manner. Our work demonstrates that topic modeling is well suited for iCBT research and has potential to expose clinically relevant information in vast text data.

## Introduction

Internet-delivered cognitive behavioral therapy (iCBT) is an effective treatment for generalized anxiety disorder (GAD) [1-4]. Additionally, iCBT programs typically store data automatically, which is convenient in terms of computerized text analysis methods, or text mining. Such methods can vastly extend the scale of traditional human-based content analysis [5]. Together with increasing data availability, text mining provides opportunities for treatment personalization and may reveal mechanisms of or obstacles to behavior change. For example, a previous study analyzed texts written during an iCBT for GAD, demonstrating a covariation between negative emotion words and symptom change over the course of treatment [6].

Many of the previous studies that have used computerized methods to analyze psychotherapy texts have relied on predetermined word categories in text classification [7-9]. Word categorization tools developed for the purposes of psychological research are theory-driven and easy to interpret [10]. Nonetheless, approaches that are more data-driven might reveal textual aspects not considered in theory-driven categorizations. An example of a data-driven approach applicable to iCBT research is topic modeling.

Topic models, such as the latent Dirichlet allocation (LDA), are unsupervised machine learning models that reduce data dimensionality by expressing a text as a mixture of latent topics [11]. LDA has been successful in detecting meaningful topics that occur in face-to-face psychotherapy transcripts [12-14]. A recent study that applied LDA to psychotherapy session transcripts found a covariation between topics with descriptions of positive experiences and a symptom decrease whereas topics that reflected discussion about treatment were associated with a symptom increase [13]. The latter also predicted alliance rupture in therapy sessions. Thus, LDA may reveal information on how to tailor interventions and improve treatment outcomes.

Previous psychotherapy topic modeling studies have used text data from whole therapy sessions with relatively free-flowing speech. This type of data is rich and has potential to reveal a wide spectrum of contents in language use during the psychotherapy process. We argue, however, that the more structured iCBT data has some benefits. First, data with a spectrum of contents that is too wide may not be ideal in terms of exploratory statistical analysis due to a phenomenon known as “the statistical curse of dimensionality: If the data have a dimension *d*, then we need a sample size *n* that grows exponentially with *d*” [15]. Simply put, rare word combinations need very large data sets to occur frequently enough for statistical estimation, and with an increasing number of utterances, most combinations get rare. Naturalistic iCBT data accrue rapidly and pertain to a comparatively narrow language space as iCBTs generally consist of standardized assignments. Second, focusing on assignment-specific data may increase the currently lacking and sought-after understanding of the meaning of specific therapeutic components [16,17]. Compared with more traditional component studies, topic modeling assignment texts could reveal benefits or harms specific to some individuals or contents that are missed in group-level comparisons in randomized controlled trials. From a topic modeling perspective, focusing on texts derived from one predetermined therapy assignment as opposed to many helps to avoid the topic model picking up task-related variation in the language use. As the meaning of language use could be context-specific, use of one assignment should also serve the aim of finding interpretable topic-outcome associations with practical implications. Despite these beneficial aspects, iCBT data have not yet been widely used for topic modeling (for an exception, see the study by Hoogendoorn et al [18]).

Regardless of the specific application context, estimating an LDA topic model requires an analyst to specify the number of latent topics in the model. This poses a challenge when using large naturalistic data sets such as iCBT texts where it is rarely possible to predetermine the distinct semantic contents in the data. Selection of the number of topics needs to be performed with care, as too few and too many topics can both affect the reliability of LDA model estimation [19]. Previous psychotherapy studies, for example a study by Atzil-Slonim et al [13], have used heuristic methods to select topic number. This may lead to suboptimal models containing idiosyncratic topics, which in turn can reduce the comparability and performance of topic models in psychotherapy research. However, the optimal strategy for topic number selection remains an unresolved challenge in topic modeling literature. New, promising, fully data-driven methods for topic-number selection are emerging, and here we examined their potential in iCBT topic modeling [19].

In this paper, we applied topic modeling to a large, naturalistic set of text data from iCBT for GAD that is offered as a part of public health care in Finland [2]. As a central element in GAD is worry, we focused on worry diary task sheets that contain patients’ descriptions of their worrisome thoughts. The worry diary was introduced at the early stages of treatment and carried on throughout the treatment, thus offering a good representation of the patient population’s writing behaviors. As a preliminary analysis, we examined whether worry diary writing activity was associated with treatment response. Our aims were to (1) explore topic modeling in iCBT data, focusing specifically on defining the optimal number of topics, and (2) investigate associations between found topics and treatment response. We expected to find meaningful topics associated with treatment response in an interpretable way. Our findings should be useful when designing optimal psychotherapy programs and instructions for worry-diary tasks and potentially when predicting who will benefit from these tasks.

## Methods

### Data

#### Participants

The data were obtained as part of routine care from the therapist-assisted iCBT for GAD, manufactured and delivered by the HUS Helsinki University Hospital (HUS-iCBT). HUS-iCBT for GAD is a standardized treatment consisting of 12 weekly sessions and a follow-up session 3 months after treatment completion. The treatment is part of the Finnish public specialized mental health care and targets adult patients and minors aged 16 years and older with mild to moderately severe symptoms. The exclusion criteria are suicidality, acute psychosis, serious personality disorder, and neurological or neuropsychiatric disorders that affect cognitive functioning. For a more detailed description of the treatment, see Ritola et al [2].

The original data set consisted of 2218 patients who had entered and completed or dropped out of the treatment between January 2015 and September 2019. As we were interested in the actual observed per-session symptom decline, we used all available data efficiently in multilevel models (see the section Treatment Response Models). Our goal was to model the symptom change of both completers and dropouts in a naturalistic manner, and therefore we did not impute any missing data. As the patient was required to complete the symptom questionnaires to proceed within each session, complete symptom data were available from all the sessions that each patient completed.

#### Text Data

Our text data were drawn from a worry diary task sheet, used as a part of 3 different between-session assignments throughout the treatment. The assignments were (1) simple worry diary, where the patients write observations about their worries and related behaviors, (2) worry postponement by writing in the worry diary within a certain time frame during the day, and (3) practicing problem-solving skills. Patients were not required to complete the between-session assignments to proceed in the treatment, and they were free to use the task sheet as often as they wanted. For a more detailed description of the worry diary, see Multimedia Appendix 1.

#### Outcome Measure

At the beginning of each session, the patient’s anxiety symptoms were assessed using the Generalized Anxiety Disorder 7-item scale (GAD-7) [20]. The GAD-7 sum score is a suitable measure for symptom severity with good temporal measurement invariance [21,22]. We defined continuous-valued treatment response as the rate of per-session change in the GAD-7 sum scores throughout the treatment. The exclusion criterion for the study was a baseline GAD-7 score of less than 8, which is a recommended cutoff point for GAD screening [23].

### Ethics Approval

This study is a part of a research project that has received permission from the ethical board of HUS Helsinki University Hospital to use the data (approval number HUS/1861/2020).

### Topic Modeling

#### Text Preprocessing

Our text corpus consisted of the worry diary task sheet entries (Multimedia Appendix 1). We preprocessed the texts by tokenizing and stemming the words as well as removing punctuation and common stop words (common words with little meaning such as *and* or *it*). The preprocessing was performed using the R package Corpus [24]. The original data contained a limited number of entries written in English or Swedish, which were removed. For an illustrative example of data preprocessing, see Multimedia Appendix 1.

#### Latent Dirichlet Allocation

We used LDA for the topic modeling of worry diary entries [11]. LDA is a widely used probabilistic model that represents each text document as a mixture of latent topics, whereas each latent topic is defined by a distribution over the words in the corpus [25]. In our data, the corpus is the whole data set of worry diary entries, whereas each entry written by a patient is a document. Each worry diary entry *i* is given an estimate θ_ik_ that represents the probability of topic *k* occurring in that entry. This numeric representation of latent topics in diary entries can then be used to associate writing about each topic with the treatment outcome. We used the R package textmineR to compute the LDA models [26]. For technical details and an illustrative example of the LDA model, see Multimedia Appendix 1.

#### Selection of Number of Topics

As noted, the selection of the number of topics *k* is important because it affects a reliable estimation of the posterior LDA distribution and thus the generalizability of observed associations with treatment response. We aimed to solve the problem by using a Bayesian approach, where the data dictate the desirable parameter value, as suggested by the original work by Chen and Doss [19]. For a more detailed description of the topic number selection procedure, see Multimedia Appendix 1. Essentially, the procedure controls for overfitting to data according to Bayesian model selection principles.

The amount of available data can affect how a complex model—how large a value for *k*—is found using a data-driven approach. Therefore, and to better understand the effects of the choice for *k*, we formed an additional LDA model using a heuristic selection process that emphasizes the interpretability of the resulting topics. That is, we aimed for topics that were semantically coherent, distinguishable from each other, and easy to identify from the texts. In this approach, we ran LDA models with *k* starting from 10, increasing it by intervals of 5 until the interpretability no longer continued to improve when more topics were added.

### Correlates of Treatment Response

#### Data Set for Modeling

The patients who had zero worry diary entries were not included in the LDA modeling corpus. To model the full range of writing activity, we included these patients in the multilevel modeling data set. For the interpretability of the treatment response effect sizes, we standardized the GAD-7 sum scores according to the baseline GAD-7 measurements. To facilitate the interpretation of the model intercepts as anxiety at the beginning of the treatment, we set the running number of therapy sessions to start from 0.

To model writing activity, we defined 4 variables for the number of worry diary entries. The first one was the total number of entries throughout the treatment, labeled as total entries. We then divided the number of entries according to the different worry diary task assignments and created 3 additional variables for the number of entries: entries 1 (worry diary), entries 2 (worry postponement), and entries 3 (problem solving).

To use the topics from the LDA models as correlates of treatment response, we assessed the average occurrence of a topic for each patient by calculating the mean value of the LDA model’s topic probability parameter θ over the patient’s worry diary entries. For those patients with no entries, the occurrence of each topic was set at 0.

#### Treatment Response Models

We defined 2 baseline treatment response models by including the session number as a fixed-effect covariate and the within-patient time-average level of anxiety as a random intercept [27]. Model 0 included random intercept only, whereas model 1 also included a within-patient random slope. Both models were adjusted for age and sex. We compared model 0 and model 1 using a likelihood ratio test, and the model with better fit was selected as the base model for additional correlates. For assessment of the treatment-response moderator effect, all following models included a correlate-by-session interaction.

We estimated the association of the worry diary writing activity with treatment response using 2 separate models. Model 2 included the total entries as a fixed-effect correlate. In model 3, we used the 3 other entry variables as fixed-effect correlates to estimate the effects of the different worry diary task assignments.

We then estimated the association of the topics from the 2 LDA models with treatment response. Each topic was separately added as a fixed-effect correlate to the base model (model 4). We then adjusted the models for the 3 entry variables to separate the independent association of a topic with treatment response from its association with writing activity under different assignments (model 5). Finally, the topics with a significant treatment-response effect were additionally adjusted with other significant topics within the same LDA model to account for potential confounding effects of topics with each other (model 6). All analyses were conducted using R (version 3.6.3, R Foundation for Statistical Computing) [28]. R code equations for the models are presented in Multimedia Appendix 1.

## Results

### Patient Characteristics

Table 1 presents the descriptive characteristics of our data. After data preprocessing, the final LDA modeling corpus consisted of 11,897 worry diary entries. In the multilevel modeling sample, the number of diary entries per patient varied between 0 and 97. Those patients with 0 diary entries were on average younger, had finished fewer sessions, were much less likely to complete all treatment sessions, and were more likely men.

**Table 1:** Baseline characteristics after data preprocessing.

			Multilevel modeling data set (n=1686^a^)		LDA^b^ modeling corpus (n=1448)		Patients with 0 entries (n=239)
**Gender, n (%)**							
	Female	1322 (78)		1155 (80)		165 (69)	
	Male	364 (22)		239 (20)		74 (31)	
Age (years), mean (SD)			33.2 (12.0)		33.5 (12.0)		31.7 (11.7)
Number of finished sessions, mean (SD)			7.8 (4.4)		8.6 (4.0)		3.2 (3.2)
Finished all 12 treatment sessions, n (%)			730 (43)		712 (49)		16 (0.1)
**Number of diary entries, mean (SD)**			7.1 (8.7)		8.2 (8.9)		0
	Entries 1 (worry diary)	4.7 (5.3)		5.5 (5.4)		0	
	Entries 2 (worry postponement)	1.6 (4.2)		1.9 (4.5)		0	
	Entries 3 (problem solving)	0.7 (1.6)		0.9 (1.7)		0	
GAD-7^c^ at beginning of treatment, mean (SD)			13.1 (3.6)		13.1 (3.6)		13.2 (3.4)

^a^Patients with missing background data (n=11) or GAD-7 score < 8 (n=521) were excluded.

^b^LDA: Latent Dirichlet allocation.

^c^GAD-7: Generalized Anxiety Disorder–7 item.

### LDA Models

We selected 7 as the optimal number of topics for the data-driven model using the Bayesian approach for topic number selection (Multimedia Appendix 1, Figure S3 and Table S1). For the interpretability-based model, we selected 25 as the optimal number of topics. For descriptions of the full LDA models, see Multimedia Appendix 1.

### Continuous-Valued Treatment Response Model

There was heterogeneity in symptom trajectories between patients as indicated by the better fit of model 1 with a random slope compared with model 0 (random intercept only; χ^2^_2_=760.17; *P*<.001). Thus, model 1 was selected as a base model for additional correlates. In model 1, there was a significant association between session number and anxiety symptoms (B=–0.14; 95% CI –0.15 to –0.13). That is, the GAD-7 score declined on average 0.14 standard deviations in each treatment session. The variability between patient symptom trajectories was large, as indicated by the random slope standard deviation of 0.1.

### Writing Activity as a Correlate of Continuous-Valued Treatment Response

In model 2, the total number of worry diary entries had a significant treatment-response moderator effect (B=0.001; 95% CI 0.000 to 0.002; for session-by-entry interaction). The effect size was modest: an increase of 1 entry in the total number of entries was associated with a 0.001 standard deviation slower than average decline in anxiety. In model 3, only the number of entries written during later phases of treatment remained significant treatment-response moderators (task assignments worry postponement and problem solving; Table 2). A larger number of entries written during the first task assignment was associated with on average more severe baseline anxiety, as reflected by the baseline effect of entries 1 on anxiety (Table 2).

**Table 2:** A multilevel regression model associating continuous-valued treatment response with writing activity during different worry diary task assignments (n=1686). Number of observations (GAD-7 measurements)=13,205.

Effect			Estimate^a^		95% CI		*P* value
**Fixed effects**							
	Intercept	−0.087		−0.260 to 0.087		.33	
	Session number	−0.147		−0.158 to −0.135		<.001	
	Treatment moderation effect, entries 1 (change/session)^b^	−0.001		−0.002 to 0.001		.23	
	Treatment moderation effect, entries 2 (change/session)	0.002		0.000 to 0.003		.03	
	Treatment moderation effect, entries 3 (change/session)	0.005		0.001 to 0.010		.02	
	Baseline effect, entries 1	0.014		0.004 to 0.023		.006	
	Baseline effect, entries 2	−0.003		−0.017 to 0.010		.61	
	Baseline effect, entries 3	−0.031		−0.066 to 0.004		.08	
	Age	−0.009		−0.013 to −0.005		<.001	
	Sex	0.086		−0.029 to 0.201		.14	
**Random effects**							
	Residual variance	0.64		—^c^		—	
	Between-patients intercept standard deviation	0.90		—		—	
	Between-patients slope standard deviation	0.10		—		—	
	Intercept-slope correlation	−0.15		—		—	

^a^Estimate: regression coefficient.

^b^The interaction between session number and each entry variable was interpreted as the treatment moderation effect.

^c^Not applicable.

### Latent Topics as Correlates of Treatment Response

Both of the LDA models contained 2 topics that moderated treatment response. For descriptions of those topics, see Table 3.

**Table 3:** Topics in the latent Dirichlet allocation models that moderated treatment response.

Model and topic			Top 10 words^a^		Interpretation of content^b^		Example^c^
**7 topic model**							
	1	child, car, how, son, father, dog, husband’s, home, mother, son’s		Well-being of family, pets, and loved ones		“What if my father gets in a car accident?”	
	4	self, thing, thoughts, life, mind, things, try, feeling, own, only		Monitoring of thoughts and worries		“Once again I am thinking about all the things that are wrong in my life.”	
**25 topic model**							
	21	write, internet therapy, therapy, worry, worry diary, write/book/letter, message, assignment, this, part		Internet therapy		“I’m afraid that the internet therapy does not work for me.”	
	24	child, father, mother, how, mother’s, husband’s, son, daughter, children’s, child		Children, family issues		“Got into an argument with my husband about taking our daughter to daycare.”	

^a^Words are translated from the Finnish language and appear on descending order based on their word-topic probability in the latent Dirichlet allocation models.

^b^Interpretation of content is based on a qualitative inspection of diary entries with a strong representation of each topic.

^c^Examples are generated by the first author and based on typical diary entries representing each topic.

#### Data-Driven Model

**Figure 1:**
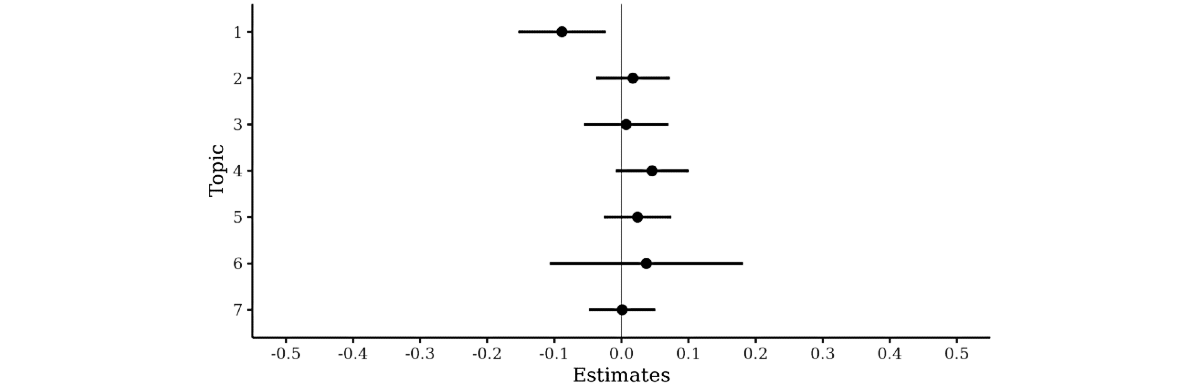
Topics from the data-driven latent Dirichlet allocation model as moderators of treatment response, adjusted for the writing activity during different worry diary task assignments.

Topic 1, which was interpreted as worries about the well-being of family and loved ones, was associated with a faster than average per-session decrease in anxiety (B=–0.10 SD/session/∆θ; 95% CI –016 to –0.03). That is, on average, a hypothetical patient who only wrote about topic 1 (mean topic probability θ for topic 1=1) recovered 0.1 GAD-7 standard deviations faster per session as compared with a patient who never wrote about the topic (mean topic probability for topic 1=0). The observed range of mean topic probability for this topic was from 0.0007 to 0.90. Topic 4 (monitoring of thoughts and worries) was associated with a slower than average per-session decline in anxiety (B=0.06 SD/session/∆θ; 95% CI 0.01 to 0.11). After adjusting for the number of entries during the different task assignments, only topic 1 remained a significant moderator of treatment response (Figure 1). Topic 1 also remained a significant moderator when topics 1 and 4 were adjusted with each other.

#### Interpretability-Based Model

**Figure 2:**
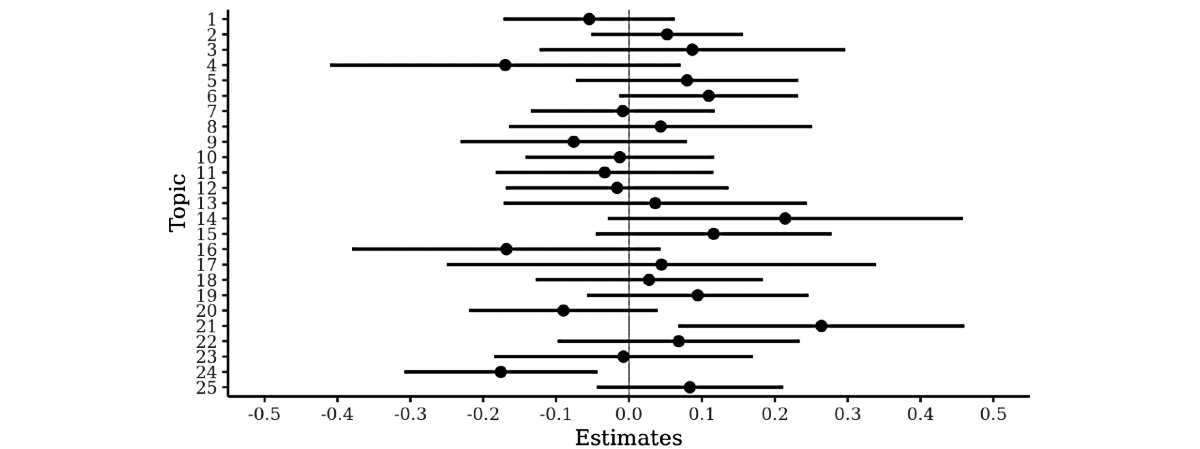
Topics from the interpretability-based latent Dirichlet allocation model as moderators of treatment response, adjusted for the writing activity during different worry diary task assignments.

Topic 21 (internet therapy) was associated with a slower than average per-session decrease in anxiety (B=0.27 SD/session/∆θ; 95% CI 0.07 to 0.46), whereas topic 24 (children, family issues) was associated with faster than average decrease in anxiety (B=–0.18 SD/session/∆θ; 95% CI –0.31 to –0.05). Both topics remained significant moderators of the treatment response after adjusting for the number of entries during the different task assignments (Figure 2). Finally, both topics remained significant moderators of the treatment response when their treatment effects were adjusted with each other.

## Discussion

### Principal Results

In this study, we used topic modeling to analyze text data from worry diary entries written during iCBT treatment for GAD. Higher worry diary writing activity toward the end of the treatment was weakly associated with worse treatment response, defined as a slower per-session symptom change. Our topic models successfully extracted meaningful topics from iCBT texts, some of which were associated with treatment response in an interpretable manner. This is in line with previous psychotherapy topic modeling research [13,18]. Our results extend the previous work by demonstrating topic modeling to be suitable for iCBT task-specific data.

### Topics and Their Associations With Treatment Response

Both LDA models contained a topic that reflected worrying about other people and was associated with a faster than average symptom decrease. For the data-driven model, this topic was labeled “well-being of family, pets, and loved ones,” based on the contents of entries that were representative of that topic. For the interpretability-based model, this topic was labeled “children, family issues” because it focused more narrowly on worries pertaining to close family. Ruminative self-focus has been associated with depression, anxiety, and negative emotionality [29-31]. Thus, worrying about others rather than merely ruminating about one’s self-related problems could reflect a healthy attention to the surrounding world. However, worries considering family members or other important characters may also indicate the presence of important relationships in a patient’s life, whereas patients suffering from social isolation would be less likely to write about these topics. Thus, our finding is also in line with research that associates social support with better treatment success, whereas loneliness and a lack of social support are associated with worse outcomes [32,33].

The data-driven model contained another topic that moderated treatment response, labeled “monitoring of thoughts and worries,” which was associated with worse treatment response. The entries that represented this topic were typically descriptions of a patient’s recurrent focus on worries, appearing as representations of ruminative self-focus [30]. Thus, our finding is in line with the results of a recent meta-analysis that reported the severity of posttreatment anxiety to be associated with higher levels of repetitive negative thinking in the form of rumination or persistent worry [34].

After controlling for the overall writing activity, however, the aforementioned moderation effect related to thought monitoring was no longer significant. A greater total number of worry diary entries was associated with a slower than average symptom decrease, which was explained by the writing activity in the later phases of treatment. This indicates that the occurrence of the “monitoring of thoughts and worries” topic may be associated with high levels of writing activity that continue into the late phases of treatment. In other words, late-phase highly active writers seem to include a group of patients who are not optimally benefitting from the treatment but exhibit persistent worry monitoring behavior. This could partially explain the counterintuitive association between higher writing activity and worse treatment response.

Besides potentially indicating persistent anxiety, the association of high writing activity with the “thought monitoring” topic could also have to do with task-related issues. For example, some patients might have difficulties in adhering to the worry postponement task, which could result in many ruminative entries when the number of entries should be limited. It is also possible that for some patients, the use of a worry diary in the beginning of the treatment could lead to an increased attention to worries and thus lead to a vicious circle of rumination. In any case, our results suggest that what the patients write might be more meaningful than how much they write, supporting the view that the quality of psychotherapy homework completion is meaningful when assessing homework-outcome relations [35]. Furthermore, our findings demonstrate that using topic modeling alongside other correlates (such as writing activity) can offer a broader understanding of treatment effect moderators as compared with using either of them separately.

The interpretation-based model also contained another topic that moderated treatment response labeled “internet therapy.” This was associated with a worse treatment response. This topic was often associated with complaints about the treatment or worries regarding the helpfulness of the treatment. Our finding is in line with Atzil-Slonim et al [13], who reported that treatment-related topics were associated with alliance ruptures and worse treatment outcomes. As these complaints can be easy to identify from iCBT texts, our finding may have applicable value in recognizing patients who are not on track in terms of recovery and may need additional support [36].

When defining the topic model, we specifically focused on defining an unbiased number of topics by adopting a data-driven selection method with a Bayesian approach [19]. The resulting data-driven model consisted of 7 topics. By comparison, our additional interpretability-based model with number of topics selected using a heuristic approach consisted of 25 topics. Based on our qualitative inspection of the worry diary texts that represented topics from the models, the topics in the interpretability-based model appeared easier to identify from the texts and were more semantically coherent than the topics from the data-driven model. The interpretability-based model also included a more diverse range of topics, offering a broader perspective on the contents that appeared in the diary texts.

However, our interpretability-based model also contained some idiosyncratic topics that strongly reflected the writings of one or few patients (Multimedia Appendix 1, Tables S4 and S5). As topic models are descriptive in nature, the idiosyncratic topics do not pose a problem per se. It has been argued that allowing some idiosyncratic topics in a topic model can be useful to separate meaningful or representative topics from “noise” in the data [13]. However, if the topic model is thought to represent a broader patient population, the idiosyncratic topics can be misleading. Furthermore, the idiosyncrasies need to be taken into account when associating the topics with treatment outcomes. In conclusion, neither approach for topic number selection was unambiguously better in our data; rather, both had benefits and drawbacks that should be considered. However, we observed some robust correlates of treatment response across the 2 very different representations.

### Strengths and Limitations

The strengths of our study include the nature of our data, which were derived from a naturalistic and nationwide setting of iCBT offered as a part of national public health care. Thus, our data likely constitute a representative sample from the target patient population. Our data set was also fairly large in terms of individual patients, improving the generalizability of our results when compared with previous studies using topic modeling to predict outcomes in samples of under 100 patients [13,18]. Our text corpus used in topic modeling consisted of entries on a task sheet targeted for writing about worries. This type of data offers precise information compared with, for example, data from whole psychotherapy sessions.

Despite our large sample size in comparison with previous research, it is nonetheless a rather moderately sized data set for machine learning. In terms of topic modeling, the amount of text produced per patient in our study was small compared with the previous study by Atzil-Slonim et al [13] that used whole therapy transcripts. Furthermore, it must be noted that the direction of causality cannot be determined from our models. For example, certain topics’ association with symptom change may be due to usefulness of writing about that topic or it may reflect patient functioning.

### Future Research

As discussed above, the topic number selection method has effects on the topic model estimation. Future iCBT topic modeling research should be mindful about these effects on the quality and generalizability of topics and their associations with treatment outcomes. Furthermore, our study demonstrated topic modeling to be practical and informative when using worry diary texts from an iCBT for GAD. In the future, topic modeling could be used in research on different disorder-specific or transdiagnostic iCBT programs. Topic modeling could also present a means to examine and compare the relative importance and meaning of different text-based tasks within or across different treatment programs, which could offer valuable information in terms of treatment development.

### Conclusions

This study demonstrated that topic modeling is a suitable and practical research method for iCBT data. We found topics from a single recurring worry diary task from an iCBT for GAD that were associated with treatment outcomes. Writing about worries regarding people close to the patient was associated with better treatment response. In contrast, monitoring of worries and worries concerning the treatment were associated with worse treatment response. This type of content information has potential for practical implications, such as in informing clinicians about the meaningful patterns in their patients’ writing behaviors. The topics also rendered other research variables, such as patients’ writing activity, more interpretable.

## Appendix

Multimedia Appendix 1 Supplementary material describing topic modeling procedure, multilevel model equations and topic modeling results in detail.

## Abbreviations

GAD-7: Generalized Anxiety Disorder–7 item
HUS-iCBT: internet-delivered cognitive behavioral therapy manufactured and delivered by the HUS Helsinki University Hospital
iCBT: internet-delivered cognitive behavioral therapy
LDA: latent Dirichlet allocation
