# Supplementary material for: Text Topics and Treatment Response in Internet-Delivered Cognitive Behavioral Therapy for Generalized Anxiety Disorder: Text Mining Study
Source: J Med Internet Res. 2022 Nov 9;24(11):e38911. doi: 10.2196/38911 (PMC9685509; doi:10.2196/38911)
Supplement: Multimedia Appendix 1 [file jmir_v24i11e38911_app1.docx]

Online Appendix

Text Topics and Treatment Response in Internet-Delivered Cognitive Behavioral Therapy for Generalized Anxiety Disorder: Text Mining Study

This is a Multimedia Appendix to a full manuscript published in the J Med Internet Res. For full copyright and citation information see http://dx.doi.org/10.2196/38911

Contents

[The Worry Diary and Preprocessing of Text Data 1](#_Toc100760043)

[Latent Dirichlet Allocation 2](#_Toc100760044)

[The Data-driven Topic Number Selection Method 3](#_Toc100760045)

[Treatment Response Models 4](#_Toc100760046)

[The LDA Models 6](#_Toc100760047)

[Exploration of Distributions of Topics Among Patients 9](#_Toc100760048)

[Appendix references 11](#_Toc100760049)

# The Worry Diary and Preprocessing of Text Data

The worry diary was used between sessions throughout the treatment. That is, the patients used the worry diary task sheet in the iCBT online platform to practice the skills they had learned during the treatment sessions. The structure of the worry diary is presented in Figure S1.

The worry diary task sheet was used as a base of three distinct between-sessions assignments. After the first session, the patients were instructed to use the task sheet as a simple worry diary, that is, to write their observations on their worries and worry behaviors. The patients were reminded to use the worry diary after the second session. After the third session, the worry diary was re-introduced with instructions on practicing worry postponement by writing in the worry diary within a certain time frame during the day. This was reminded after the fifth and sixth sessions. After the tenth session, the worry diary was once again re-introduced. This time, the patients were instructed to practice problem-solving skills for their current worries. The patients were not required to complete the between-sessions assignments to proceed in the treatment. Furthermore, the patients were free to use the task sheet as often as they wanted throughout the treatment, and the diary platform was open for use for 30 days after the final treatment session. Thus, the number of entries produced in the worry diary varied between patients.

Supplementary figure 1 also illustrates the text preprocessing procedure. In short, we combined the text fields in each worry diary entry to define a text “document” for the latent Dirichlet allocation (LDA) model. We omitted the feelings-field because it contained only limited between-patients variability and mixed qualitative and quantitative information.


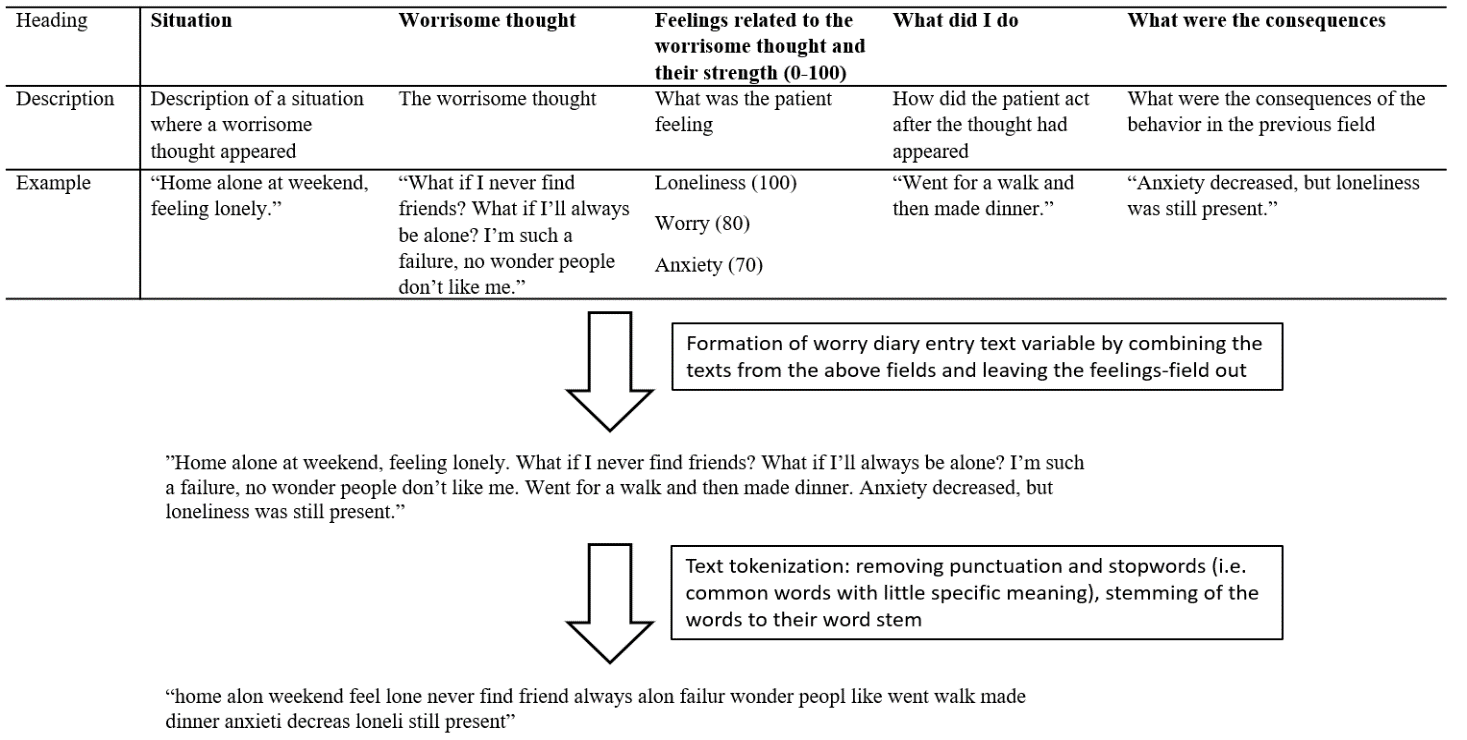


Figure S1. The worry diary task sheet and description of the text preprocessing procedure.

# Latent Dirichlet Allocation

Panel A Figure S2 illustrates the process, where the LDA algorithm defines the parameters θ and ϕ which in turn define the posterior distribution of topics in documents and words in topics, respectively. Each θ_ik_ represents the probability of a given topic *k* occurring in a document *i*. In our case, the “document” is a worry diary entry. For each entry, the sum of the topic-document probability parameter θs across all topics is 1. That is, if an entry represents a topic with a probability of 0.7, the same entry represents all other topics in that model with a probability of 0.3 in total. Accordingly, each ϕ_kn_ represents the probability of a given word *n* to represent topic *k*.

Panel B in Supplementary figure 2 describes how the topics from an imaginary three topic LDA model are represented in a worry diary entry. An entry that contains multiple words that are strong representations of a topic, is allocated with a large value for topic probability parameter θ for that topic.

A

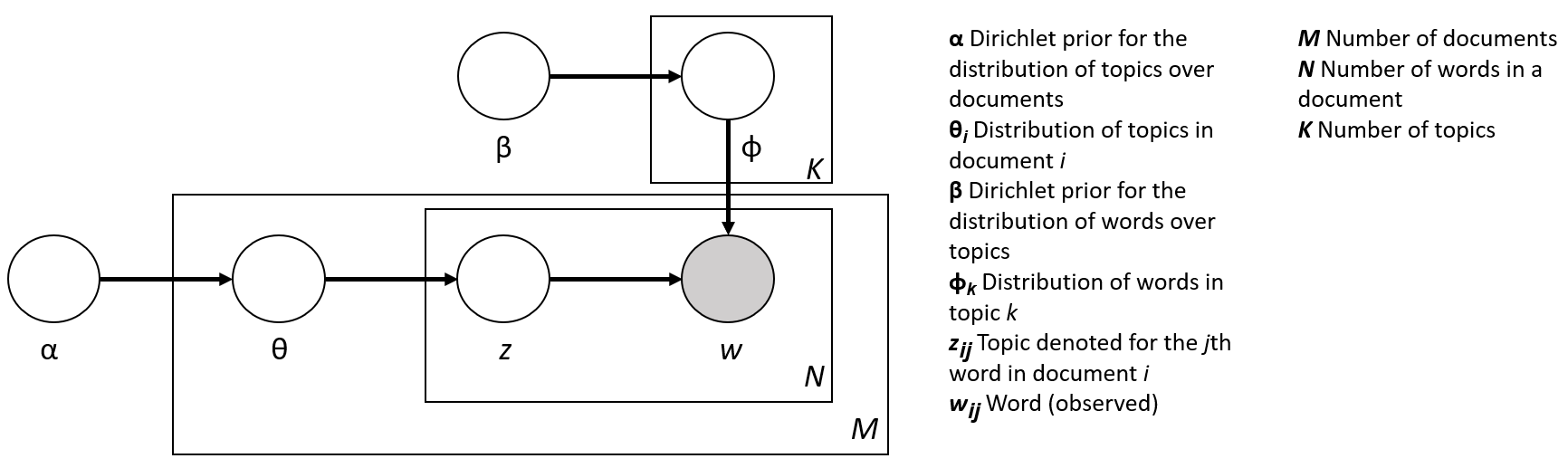


B


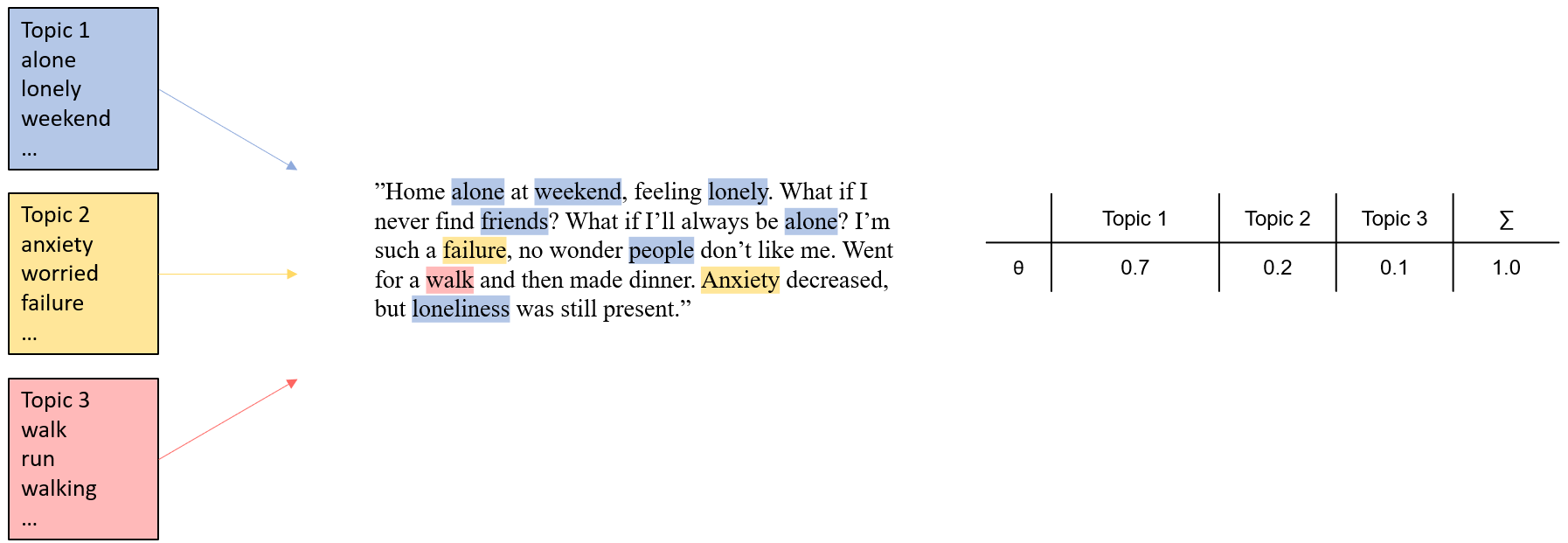


*Figure S2*. A) Graphical representation of the LDA model, adapted from Blei et al., 2003 [11]. The shaded circle represents observed words, whereas the white circles represent latent variables. The boxes represent replication of each latent variable according to number of documents, words or topics. B) Illustrative example of topics in an LDA model and how they are represented in a worry diary entry. For clarity, the text is presented in an unprocessed form.

Besides other assumptions, the LDA model is defined by a predetermined number of topics *k* and two hyperparameters: α defines the Dirichlet prior for the distribution of topics over documents, and β defines the Dirichlet prior for the distribution of words over topics (Panel A Figure S2). We used the R package ‘textmineR’ to compute the LDA model using the default values of 0.1 for initializing α, and 0.05 for β, which are commonly used values for these hyperparameters [26]. In model definition, we set the number of Gibbs sampler iterations to 2,000 and allowed for an optimization of α every 10 iterations, thus allowing for an asymmetric posterior topic-document distribution.

# The Data-driven Topic Number Selection Method

The number of topics *k* needs to be predetermined in the LDA model. One alternative for solving the problem of selecting the optimal *k* is a Bayesian approach by setting up a prior distribution over multiple possible *k*’s when defining the LDA model and then solving for the posterior distribution of *k*. In the Bayesian approach, the data dictate the desirable parameter value. However, the use of a Bayesian approach to select topic number has been hindered by the computational challenges in defining the posterior distribution for *k* due to the complexity of the LDA distributions. Recently, a possible solution for this issue was presented in the form of a pseudo-marginal Metropolis-Hastings algorithm (PMMH) which can be used to *approximate* the posterior distribution of *k*  [19]. In essence, the PMMH algorithm is a Markov chain based method that is well suited for simulations of distributions with high dimensions. For a more technical presentation, including equations for the adaptation of the PMMH algorithm for LDA, we suggest interested reader to explore the thorough description in Chen & Doss [19].

After distribution approximation, the optimal number of topics is defined by the most probable posterior value for *k*. That is, the value where most of the posterior distribution’s mass is concentrated at should reflect the number of topics with most stable fit to the data. We used the PMMH algorithm following the work by Chen & Doss [19] and the “lda.pmmh” R package and R code offered as a supplementary material of their original article. We defined the prior distribution of *k* as a uniform distribution ranging from 4 to 50 and assigned the PMMH algorithm an initial *k* of 20 (that is, the starting point for the Markov chains in the PMMH algorithm). These initial values were selected to achieve a reasonable number of topics without excessive overlap, and because a previous study using internet therapy texts used k of similar magnitude that resulted in topics with predictive value [5]. Figure 3 and Table S1 illustrate the posterior distribution for *k*. Based on the results, we chose the optimal data-driven value for *k* as 7.


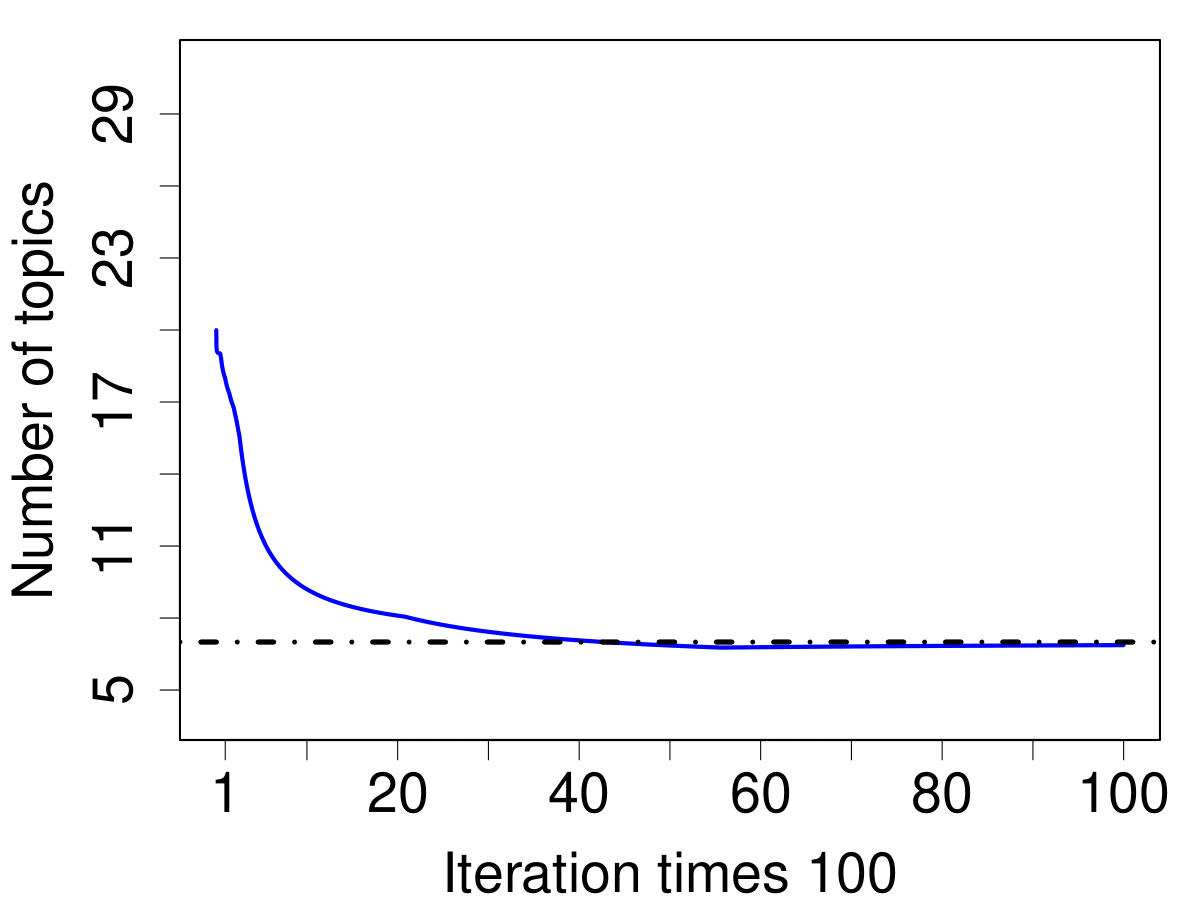


Figure S3. Running mean for value of topic number k as suggested over 10 000 iterations of posterior distribution estimation by the PMMH algorithm. The dashed line is set at 7 for reference.

| *k* | = | > | < |
| --- | --- | --- | --- |
| 5 | 0 | 100 | 0 |
| 6 | 34.84 | 65.16 | 0 |
| 7 | 62.56 | 2.60 | 34.84 |
| 8 | 0.03 | 2.57 | 97.40 |

Table S1. Concentrations of mass in posterior distribution for number of topics k estimated with the PMMH algorithm. 62.56 % of the posterior distribution’s mass was concentrated at k = 7.

# Treatment Response Models

The multilevel models for treatment response and its correlates were constructed as follows, presented as defined when using the R package “lme4”.

Treatment response according to treatment session, or the base models

Model 0
GAD-7 ~ Session + Age + Sex + (1|Patient)

Model 1
GAD-7 ~ Session + Age + Sex + (Session|Patient)

Model 0 included a patient level random intercept only, whereas Model 1 also included a patient level random slope. The regression coefficient for Session reflected the per-session change in GAD-7 sum scores throughout the treatment.

Writing activity as a correlate of treatment response

Model 2
GAD-7 ~ Session*Total entries + Age + Sex + (Session|Patient)

Model 3
GAD-7 ~ Session*Entries1 + Session*Entries2 + Session*Entries3 + Age + Sex + (Session|Patient)

The interaction between the Entries-variables and Session was interpreted as the treatment response moderation effect of writing activity. The Session1-3 variables represent entries written during different worry diary task assignments, as descripted in the sections “Text data” and “Dataset for modeling” of the manuscript and the section “The Worry Diary and Preprocessing of Text Data” of this supplement.

Topics as correlates of treatment response

Model 4
GAD-7 ~ Session*Topic + Age + Sex + (Session|Patient)

Model 5
GAD-7 ~ Session*Topic + Age + Sex + Entries1 + Entries2 + Entries3 + (Session|Patient)

Model 6
GAD-7 ~ Session*Topic + Age + Sex + Entries1 + Entries2 + Entries3 + Topic^a^ + (Session|Patient)

The interaction between the Topic and the Session was interpreted as the treatment response moderation effect of that topic. Models 4 and 5 were run for every topic from both LDA models. Model 6 was run for every topic that was a significant treatment-response moderator in Model 4, and the moderator effect was adjusted for the occurrence of other significant topics within the same LDA model (Here, the adjusting Topic variable is denoted with ^a^)

# The LDA Models

Tables S2 and S3 present the topic in the data-driven and in the interpretability-based LDA models, respectively. In the data-driven model, the most prevalent topic was Topic 5, with a mean topic-document probability θ of 0.20 over all the entries. That is, the words in a randomly chosen entry represented Topic 5 with a 20% chance, but the entry could also contain other topics. The least prevalent topic was Topic 6, with a mean θ of 0.037. In the interpretability-based model, topic 6 was the most prevalent topic with a mean θ of 0.071. Topic 14 was the least prevalent topic with a mean θ of 0.018.

| Topic | Top 10 words | Interpretation of content | Example |
| --- | --- | --- | --- |
| 1 | child, car, how, son, father, dog, husband’s, home, mother, son’s | Well-being of family, pets and loved ones | “My mother is looking after my dog. Is everything going well there.”  “What if my father gets in a car accident?” |
| 2 | friend(close), friend(casual), self, boyfriend, thing, people, every, want, alone, husband’s | Relationships, social events | “A friend is having a party, and there will be people I don’t know. What if everyone thinks I’m strange?”  “Had a fight over a small thing with boyfriend. I fear that he’ll leave me and I end up alone.” |
| 3 | thing, money, how, workplace/job, work, still, get, every, have to, can/be able to | Work and financial issues | “Haven’t heard from a job I applied to. How will my money last?”  “Got a new assignment at work. What if I cannot do it?” |
| 4 | self, thing, thoughts, life, mind, things, try, feeling, own, only | Monitoring of thoughts and worries | “Negative thoughts come to mind all the time. I would feel better if I would not focus only on myself.”  “Once again thinking about all the things that are wrong in my life.” |
| 5 | day, every, do, manage/be able to, anxious, feeling, get, again, how, time | Things that should be done | “There’s so much to do next day, will I have time to do everything?”  “I should get myself to clean my home, but I feel so anxious that I don’t think I’m able to.” |
| 6 | me, my, mine, but, then, indeed/surely, am, quite, like, me | Personal pronouns, non-specific descriptive text | “Today I had a quite good day, I was at home with my cat but then…” |
| 7 | feeling, try, again, heart, go, day, symptom, feel, anxiety, fear | Fear of sickness, physical symptoms of anxiety | “I have had a strange feeling in my feet all day again. I fear that this is a symptom of some serious disease.”  “Anxiety makes my heart race. I try to relax.” |

Table S2. Topics from the data-driven LDA model. Under the column “Top 10 words”, ten words with the largest word-topic probability φ are listed for each topic in descending order of magnitude. The words are translated from the Finnish language.
The interpretation of content is based on a qualitative inspection of diary entries with a strong representation of each topic. The examples are generated by the first author and are based on typical diary entries representing each topic.

| Topic | Top 10 words | Interpretation of content | Example |
| --- | --- | --- | --- |
| 1 | serious, symptom, fear, pain, doctor, disease, hurt, some, pains, physical | Fear of sickness | “I have had a strange feeling in my feet all day again. I fear that this is a symptom of some serious disease”  “My stomach hurts again, I should call a doctor about my pains.” |
| 2 | feeling, heart, feel, try, anxious, started, beat, anxiety, can, feels | Bodily symptoms of anxiety | “My hands shake and I feel dizzy. I fear that this feeling never goes away.”  “My heart started to beat fast and I was anxious. I tried to relax but anxiety remained.” |
| 3 | car, dog, drive(verb), home, journey, leave, husbands, cat, come, drive(noun) | Cars and pets | “Driving home, the journey is long. What if my car falls off the road?”  “I fear my dog is sick.” |
| 4 | me, my, mine, but, indeed/surely, then, am, quite, me, like | Personal pronouns, non-specific descriptive text | “Today I had a quite good day, I was at home with my cat but then…” |
| 5 | friend(casual), friend(close), people, every, alone, go, anxious, party, boyfriend, home | Relationships, social events | “A friend is having a party, and there will be people I don’t know. What if everyone thinks I’m strange?”  “Had a fight over a small thing with my boyfriend. I fear that he’ll leave me and I end up alone.” |
| 6 | life, self, everything, only, anymore, did not, feels, can, things, own | Feelings of failure | “My life stands still, everything stays the same.”  “Everyone else has someone, I myself cannot find someone my own anymore.” |
| 7 | thing, husband, message, boyfriend, said, situation, call, anymore, want, things | Conflicts in relationships | “Boyfriend does not answer my message. He doesn’t want to be with me anymore.”  “Said a thing at work that was inappropriate in that situation. I worry I made a coworker upset.” |
| 8 | on, come, door, closed, everything, go, off, fire, inside, house | Fear of something bad happening | “Did I remember to turn everything off? I fear I left the stove on and there will be a fire in the house.”  “If the door is not closed, someone could come inside.” |
| 9 | morning, sleep(noun), sleeping, sleep(verb), wake up, night, can, day, woke up, get sleep | Sleep issues | “I woke up too early in the morning and could not get sleep anymore.”  “Got too little sleep last night, how can I manage the day?” |
| 10 | feeling, day, manage/be able to, after, whole, home, again, morning, go, tired | Psychological or physical tiredness | “Feeling tired, how am I able to go on all day?”  “How long can I manage with these feelings?” |
| 11 | try, thing, thoughts, self, mind, feeling, somewhat, things, anxiety, did | Efforts to control anxiety | “Negative thoughts came to mind. Tried to focus on other things.”  “Did a relaxation exercise. Feeling of anxiety decreased.” |
| 12 | fear, anxiety, thoughts, mind, worry, day, anxious, things, own, anxiety | Worries and anxiety due to changing reasons | “Anxious thoughts came to mind, what if…”  “Fear of death/sickness/going crazy…” |
| 13 | doctor, medication, take, call, medicine, can, thing, went, have to, keep | Healthcare use, medications | “I forgot to take my medication, I wonder if I should call my doctor.”  “I have to go to a doctor’s appointment again, what if they don’t listen to me?” |
| 14 | eating, food, again, bad, eat, self, good, back, ate, hair | Eating issues, fear of gaining weight | “I caught myself eating too much food again, I will never look good.”  “I felt bad about myself after eating that chocolate bar.” |
| 15 | school, can/know how to, teacher, bad, course, enough, exam, class, get, good | Studies | “What if I fail my presentation at school? I will look bad in front of the class.”  “The exam is tomorrow, I fear I haven’t studied enough.” |
|  |  |  |  |
| Topic | Top 10 words | Interpretation of content | Example |
| 16 | baby, all, come, ear, home, away, clothing, belongings, floor, husband | Fear of contamination | “Baby’s clothing dropped on the floor. I fear that some bacteria will infect the baby.”  “A strange noise comes to my ears at home. What if there’s some form of radiation?” |
| 17 | son, boys, how, self, own, whole, also, read, home country’s, news | Fear of safety threatening events (war, terrorism, violence etc.) | “What if my son gets bullied by the older boys?”  “Read news about terrorist attacks, worried about the home country’s safety.” |
| 18 | feeling, self, feels, bad, come, again, only, came, then, feel | “Meta worry”, worrying about negative emotions and anxiety | “Why am I feeling this bad again?”  “I feel that I am again focusing too much on how I feel but I can’t help it.” |
| 19 | time, already, also, week, always, much, then, day, last, so | Time | “I haven’t written here in a long time.”  “Last day I already felt as tired as the last week.” |
| 20 | in workplace, workplace, how, to work, coworker, work, have to, get, think, in work | Work | “Got a new assignment at work. What if I cannot do it?”  “Feeling anxious about going to the workplace because of issues with a coworker.” |
| 21 | write, internet therapy, therapy, worry, worry diary, write/book/letter, message, assignment, this, part | Internet therapy | “I’m afraid that the Internet therapy does not work for me.”  “It feels stupid to write to the worry diary, besides this webpage doesn’t work well.” |
| 22 | money, bill, apartment, thing, apartment’s, have to, more, how, buy, pay | Financial issues | “I have to be able to save more money so that I can keep this apartment.”  “Paid the bills, will I have enough money to buy food?” |
| 23 | travel, go, anxious, bus, home, people, away, leave, store, everything | Situations that provoke social anxiety or panic attacks | “I fear I will get anxious in the store and people notice.”  “I have to travel by bus, what if I get anxious?” |
| 24 | child, father, mother, how, mother’s, husband’s, son, daughter, children’s, child | Children, family issues | “My mother is looking after my child. Is everything going well there.”  “Got into an argument with my husband about taking our daughter to daycare.” |
| 25 | do, all, things, should, doing, have time, time, get, day, thing | Things that should be done | “There’s so much to do the next day, will I have time to do everything?”  “I should get myself to clean my home, but I feel so anxious that I don’t think I’m able to.” |

Table S3. Topics from the interpretability-based LDA model. Under the column “Top 10 words”, ten words with the largest word-topic probability φ are listed for each topic in descending order of magnitude. The words are translated from the Finnish language.
The interpretation of content is based on a qualitative inspection of diary entries with a strong representation of each topic. The examples are generated by the first author and are based on typical diary entries representing each topic.

# Exploration of Distributions of Topics Among Patients

| Topic |  | θ > 0.7 | θ > 0.6 | θ > 0.5 |
| --- | --- | --- | --- | --- |
| 1 | Patients, N | 194 | 291 | 396 |
|  | Entries, N | 316 | 520 | 808 |
|  | From same patient, N(%) | 13(4) | 21(4) | 31(4) |
| 2 | Patients, N | 397 | 541 | 680 |
|  | Entries, N | 688 | 1076 | 1537 |
|  | From same patient, N(%) | 12(2) | 14(1) | 18(1) |
| 3 | Patients, N | 267 | 386 | 530 |
|  | Entries, N | 409 | 652 | 978 |
|  | From same patient, N(%) | 21(5) | 22(3) | 23(2) |
| 4 | Patients, N | 374 | 523 | 700 |
|  | Entries, N | 649 | 1047 | 1609 |
|  | From same patient, N(%) | 29(4) | 40(4) | 51(3) |
| 5 | Patients, N | 501 | 644 | 794 |
|  | Entries, N | 936 | 1373 | 1926 |
|  | From same patient, N(%) | 15(2) | 19(1) | 24(1) |
| 6 | Patients, N | 15 | 29 | 54 |
|  | Entries, N | 60 | 102 | 159 |
|  | From same patient, N(%) | 44(73) | 63(62) | 72(45) |
| 7 | Patients, N | 391 | 514 | 637 |
|  | Entries, N | 710 | 1017 | 1425 |
|  | From same patient, N(%) | 11(2) | 14(1) | 14(1) |

Table S4. Distributions of worry diary entries with high topic-document probability θ in each topic according to number of patients in the data-driven LDA model

| Topic |  | θ > 0.7 | θ > 0.6 | θ > 0.5 |  | Topic |  | θ > 0.7 | θ > 0.6 | θ > 0.5 |
| --- | --- | --- | --- | --- | --- | --- | --- | --- | --- | --- |
| 1 | Patients, N | 23 | 54 | 114 |  | 16 | Patients, N | 7 | 16 | 38 |
|  | Entries, N | 23 | 61 | 137 |  |  | Entries, N | 10 | 24 | 58 |
|  | From same patient, N(%) | 1(4) | 4(7) | 5(4) |  |  | From same patient, N(%) | 4(40) | 5(21) | 9(16) |
| 2 | Patients, N | 24 | 60 | 124 |  | 17 | Patients, N | 1 | 13 | 26 |
|  | Entries, N | 29 | 81 | 179 |  |  | Entries, N | 1 | 13 | 31 |
|  | From same patient, N(%) | 4(14) | 5(6) | 7(4) |  |  | From same patient, N(%) | 1(100) | 1(8) | 2(6) |
| 3 | Patients, N | 8 | 21 | 46 |  | 18 | Patients, N | 6 | 15 | 40 |
|  | Entries, N | 11 | 27 | 62 |  |  | Entries, N | 45 | 84 | 137 |
|  | From same patient, N(%) | 4(36) | 6(22) | 9(15) |  |  | From same patient, N(%) | 22(49) | 36(43) | 52(38) |
| 4 | Patients, N | 1 | 4 | 10 |  | 19 | Patients, N | 2 | 8 | 29 |
|  | Entries, N | 22 | 50 | 68 |  |  | Entries, N | 2 | 8 | 35 |
|  | From same patient, N(%) | 22(100) | 47(94) | 59(87) |  |  | From same patient, N(%) | 1(50) | 1(13) | 4(11) |
| 5 | Patients, N | 22 | 49 | 94 |  | 20 | Patients, N | 7 | 31 | 72 |
|  | Entries, N | 23 | 55 | 113 |  |  | Entries, N | 7 | 32 | 84 |
|  | From same patient, N(%) | 2(9) | 2(4) | 3(3) |  |  | From same patient, N(%) | 1(14) | 2(6) | 3(4) |
| 6 | Patients, N | 14 | 49 | 114 |  | 21 | Patients, N | 11 | 31 | 65 |
|  | Entries, N | 14 | 55 | 142 |  |  | Entries, N | 11 | 32 | 83 |
|  | From same patient, N(%) | 1(7) | 3(5) | 7(5) |  |  | From same patient, N(%) | 1(9) | 2(6) | 3(4) |
| 7 | Patients, N | 18 | 57 | 129 |  | 22 | Patients, N | 12 | 31 | 61 |
|  | Entries, N | 18 | 61 | 154 |  |  | Entries, N | 15 | 35 | 75 |
|  | From same patient, N(%) | 1(6) | 2(3) | 5(3) |  |  | From same patient, N(%) | 4(27) | 5(14) | 11(15) |
| 8 | Patients, N | 18 | 28 | 45 |  | 23 | Patients, N | 17 | 45 | 83 |
|  | Entries, N | 26 | 45 | 79 |  |  | Entries, N | 19 | 58 | 114 |
|  | From same patient, N(%) | 7(27) | 10(22) | 13(16) |  |  | From same patient, N(%) | 3(16) | 4(7) | 6(5) |
| 9 | Patients, N | 22 | 46 | 105 |  | 24 | Patients, N | 7 | 28 | 69 |
|  | Entries, N | 25 | 59 | 137 |  |  | Entries, N | 8 | 36 | 88 |
|  | From same patient, N(%) | 2(8) | 3(5) | 4(3) |  |  | From same patient, N(%) | 2(25) | 6(17) | 6(7) |
| 10 | Patients, N | 26 | 63 | 112 |  | 25 | Patients, N | 35 | 83 | 165 |
|  | Entries, N | 29 | 80 | 167 |  |  | Entries, N | 37 | 93 | 203 |
|  | From same patient, N(%) | 2(7) | 4(5) | 8(5) |  |  | From same patient, N(%) | 2(5) | 3(3) | 4(2) |
| 11 | Patients, N | 7 | 20 | 61 |  |  |  |  |  |  |
|  | Entries, N | 10 | 34 | 95 |  |  |  |  |  |  |
|  | From same patient, N(%) | 3(30) | 8(24) | 15(16) |  |  |  |  |  |  |
| 12 | Patients, N | 12 | 22 | 51 |  |  |  |  |  |  |
|  | Entries, N | 16 | 34 | 71 |  |  |  |  |  |  |
|  | From same patient, N(%) | 4(25) | 9(26) | 15(21) |  |  |  |  |  |  |
| 13 | Patients, N | 9 | 18 | 47 |  |  |  |  |  |  |
|  | Entries, N | 9 | 19 | 52 |  |  |  |  |  |  |
|  | From same patient, N(%) | 1(11) | 2(11) | 3(6) |  |  |  |  |  |  |
| 14 | Patients, N | 1 | 7 | 16 |  |  |  |  |  |  |
|  | Entries, N | 1 | 7 | 17 |  |  |  |  |  |  |
|  | From same patient, N(%) | 1(100) | 1(14) | 2(12) |  |  |  |  |  |  |
| 15 | Patients, N | 17 | 38 | 82 |  |  |  |  |  |  |
|  | Entries, N | 20 | 48 | 121 |  |  |  |  |  |  |
|  | From same patient, N(%) | 3(15) | 3(6) | 7(6) |  |  |  |  |  |  |

Table S5. Distributions of worry diary entries with high topic-document probability θ in each topic according to number of patients in the interpretability-based LDA model. Examples of idiosyncratic topics are highlighted in gray.
